# Sitting Baduanjin Combined With Acupoint Massage on Frailty in Patients Undergoing Maintenance Hemodialysis: Protocol for a Randomized Controlled Trial

**DOI:** 10.2196/72352

**Published:** 2025-11-21

**Authors:** Wanyu Zhao, Shurong Zhang, Hongmei Xie, Junhong Hou, Fang Wang, Jing Shi, Shasha Luo

**Affiliations:** 1 Hospital of Chengdu University of Traditional Chinese Medicine, No. 39 Shi-er-qiao Road, Chengdu, Sichuan, 610072, China, 86 028 87783047; 2 School of Nursing, Chengdu University of Traditional Chinese Medicine, Chengdu, Sichuan, China

**Keywords:** maintenance hemodialysis, acupoint massage, sitting Baduanjin, quality of life, traditional Chinese medicine

## Abstract

**Background:**

Patients undergoing maintenance hemodialysis (MHD) endure weakness due to prolonged dialysis treatment, such as the continuous decline of muscle strength in patients, which will have varying degrees of impact on human physiological, psychological, and social functions. Effective nonpharmacological interventions can improve their mental health and quality of life (QoL).

**Objective:**

This study aimed to investigate the effects of sitting Baduanjin combined with acupoint massage on improving the frailty status of patients undergoing MHD and evaluate whether it can significantly improve their physical activity, alleviate depressive emotions, and comprehensively improve their QoL.

**Methods:**

This is a randomized controlled study to be conducted in the Affiliated Hospital of Chengdu University of Traditional Chinese Medicine, comprising 114 patients to be treated using MHD. Patients who meet the inclusion and exclusion criteria will be randomly divided into 3 groups: the control group will receive only conventional hemodialysis and care, the acupoint massage group will receive acupoint massage treatment in addition to the control group treatment, and the sitting combined with acupoint massage group will receive sitting Baduanjin combined with acupoint massage and the control group treatment. We aim to comprehensively evaluate the clinical efficacy by comparing the FRAIL (Fatigue, Resistance, Ambulation, Illness, and Weight Loss) scale scores, grip strength, 10 times sit-to-stand test, Self-Rating Depression Scale scores, and QoL (Short Form Health Survey Questionnaire) scores before and after 8 weeks of treatment among the 3 groups. Statistical analysis will be conducted using the Statistical Package for the Social Sciences software (version 25.0; IBM Corporation).

**Results:**

The first participant was enrolled on August 8, 2024. As of August 2025, a total of 52 participants have been fully registered, and the intervention is currently in progress. We expect the completion of this trial to occur within the year 2025.

**Conclusions:**

The results of this study can provide a scientific basis for the future treatment of patients with asthenia in hemodialysis.

## Introduction

### Background

Maintenance hemodialysis (MHD) is the primary treatment for end-stage renal disease. It effectively removes metabolic toxins from the body, prolongs patient survival, and improves quality of life (QoL) [1]. Frailty is a nonspecific state resulting from decreased physiological reserves or multisystem dysfunction, leading to increased vulnerability and reduced stress resistance. This makes individuals more susceptible to adverse clinical events, even in response to minor external stimuli [2]. It is a prevalent and debilitating clinical syndrome that significantly affects the QoL of patients undergoing MHD treatment [3]. Frailty is primarily characterized by unintentional weight loss, diminished grip strength, slowed walking speed, reduced physical activity, and self-reported exhaustion [4]. The presence of 1 or 2 of these criteria indicates a prefrail state, while the presence of 3 or more criteria can confirm frailty. A recent survey conducted in the United States revealed a 9% prevalence of frailty among the older population, with an even higher prevalence of 44% among those classified as prefrail [5]. A Chinese study revealed that 5.6% of individuals aged ≥60 years were classified as frail, while 49.3% exhibited characteristics indicative of prefrailty [6]. A review of international studies revealed that the prevalence of prefrailty among patients undergoing hemodialysis ranged from 50% to 63%, while the prevalence of frailty itself fluctuated between 33% and 47% [7-10]. A Chinese study revealed a prevalence of 60.7% for prefrailty and 44.8% for frailty in patients undergoing hemodialysis [11,12].

These findings indicate that all age groups of adults undergoing hemodialysis exhibited a significantly elevated prevalence of frailty, exceeding that observed in the general older population. This highlights the need for increased attention to this issue [7]. As the duration of dialysis increased, the patients exhibited a progressive decline in muscle strength, a reduction in activity levels, and the development of frailty. This condition can influence a range of physiological, psychological, and social functions, as well as self-care abilities. In addition, it increases the risk of falls, rehospitalization, and mortality, thereby reducing the QoL [13,14]. Frailty was identified as an independent predictor of all-cause mortality in patients undergoing MHD for more than 5 years [15].

Previous studies link frailty in patients undergoing MHD to physical function, depressive symptoms, and QoL [16]. Prolonged dialysis causes substantial amino acid and protein loss; patients with chronic renal failure with low protein intake (insufficient for bodily needs) have reduced muscle synthesis and impaired physical function [17]. In addition, calcium-phosphorus imbalance in patients undergoing MHD reduces skeletal muscle and joint function, while prolonged sitting and lying impairs muscle strength or mass and physical activity [16]. Due to irreversible progression and long treatment, patients often have anxiety and depression. A systematic review notes a bidirectional depression-frailty relationship: patients with depression face higher frailty risk, and patients with frailty are more likely to have depressive symptoms [17]. Declines in physical function and depression fuel frailty, creating a vicious cycle that lowers QoL, raises rehospitalization and death risks [18]. However, frailty is dynamic and reversible; timely detection or management can delay or reverse it, improving prognosis [19]. Sarcopenia is the core pathological basis of frailty in patients undergoing MHD, with most having low grip strength and activity levels [20]. MHD has a strong adverse association with frailty, with evidence from multiple studies. First, frailty is highly prevalent in patients undergoing MHD: a meta-analysis of 16 studies (2446 patients) reported a pooled frailty prevalence of 41% [21]. Second, frailty significantly elevates adverse outcomes in patients undergoing MHD. A systematic review including 23 studies (10,333 patients) showed patients with frailty undergoing MHD had higher all-cause mortality and all-cause hospitalization [22]. Third, specific factors mediate this association: xerostomia and poor nutrition correlate with frailty, jointly explaining 45.6% of frailty variance in older patients undergoing MHD [23]; age, hypoalbuminemia, depression, and malnutrition are independent frailty predictors in patients undergoing MHD [24]. This underscores frailty as a key clinical concern requiring attention in the management of patients undergoing MHD management. The prevalence of prefrailty is notably high among patients undergoing MHD. Prefrailty, an early and reversible condition between nonfrailty and frailty, can lead to adverse outcomes such as increased unplanned hospital admissions and a higher risk of other chronic diseases [24].

Early identification and treatment of frailty in patients with myocardial hypertrophy are critical for the management of this condition. Baduanjin, a low-to-moderate-intensity aerobic exercise, is based on traditional Chinese medicine (TCM) theories and integrates the yin-yang and meridian theories. Baduanjin comprises standing and sitting forms, distinguished by their gentle and slow movements. These are characterized by simplicity, ease of learning, safety, efficacy, and the absence of venue and time restrictions [25]. The practice of sitting Baduanjin has many beneficial effects, including regulation of the body, breath, and mind, as well as harmonization of the 5 viscera [26]. Furthermore, it mitigates the risk of falls and other mishaps resulting from muscle atrophy and diminished endurance, rendering it a suitable choice for patients with frailty undergoing MHD. Modern research has demonstrated that Baduanjin exhibits beneficial regulatory effects on multiple systems of the body, not only resisting aging but also significantly improving limb muscle strength, alleviating anxiety and depression, improving fatigue states, relieving pain, enhancing cardiopulmonary function, and, with long-term practice, strengthening the body and improving disease resistance [27-29].

Baduanjin can significantly improve sleep quality, alleviate anxiety and depression, and improve fatigue in patients undergoing MHD [29]. Another study indicated that 5-element music combined with Baduanjin helps reduce fatigue and alleviates frailty in patients undergoing MHD, improving the QoL [30]. From the perspective of TCM, frailty in MHD is mainly caused by a deficiency in organ functions, qi, blood, yin, and yang, with prolonged deficiency leading to fatigue. The core pathology involves spleen and kidney deficiencies. Sitting Baduanjin can harmonize qi and blood, balance yin and yang, and coordinate organ functions to achieve a balanced state, thereby improving physiological functions.

Furthermore, TCM emphasizes the overall regulatory role of the meridian system. Studies have demonstrated that acupoint massage can improve limb function, negative emotions, sleep quality, and fatigue [31,32]. Studies using acupoint massage on patients undergoing hemodialysis revealed significant improvements in fatigue and QoL with specific acupoints, including Zusanli (ST36), Sanyinjiao (SP6), Taixi (KI3), Yongquan (KI1), and Neiguan (PC6) being particularly effective [33].

### Current Research Gap

Although studies have explored the effects of Baduanjin and acupoint massage on patients undergoing MHD, there are still knowledge gaps. Most studies focus on improving specific indicators, but there is a lack of assessment of the patients’ frailty status. Baduanjin improves muscle function of patients undergoing hemodialysis, reduces systemic inflammation, and enhances skeletal muscle–related indices [34]. Massage relieves lower limb cramps [34] and boosts sleep quality via hot stone therapy [35]. Yet, massage only targets single symptoms (no effects on muscle mass and inflammation); Baduanjin needs nutritional support, covers a few frailty symptoms, and has limited skeletal muscle benefits [34,36]. Their combination could comprehensively address frailty, synergistically protect muscles, and improve frailty via multiple mechanisms. Regarding the effectiveness of combining sitting Baduanjin exercise and acupoint therapy in improving the frailty of patients undergoing MHD, there is also a lack of high-quality evidence.

### Unique Contributions of This Study

This study aims to address these research gaps through a randomized controlled trial that systematically evaluated the effects of combining sitting aduanjin with acupoint massage on frailty in patients undergoing MHD treatment. This study makes several contributions to the field. First, it is the first to combine the sitting form of the 8-section brocade with acupoint massage to provide an 8-week comprehensive nonpharmacological intervention strategy with long-term follow-up. A systematic approach is used in this study to evaluate numerous indicators in patients undergoing MHD, including frailty, grip strength, lower limb function, depression status, and QoL. The findings contribute to the evidence base for effective and high-quality nonpharmacological interventions for patients with frailty undergoing MHD.

### Purpose and Significance

This study aims to investigate the effects of various interventions on frailty. The primary objective is to investigate whether combining sitting Baduanjin with acupoint massage could improve frailty in individuals with mild-to-moderate heart disease, reduce muscle atrophy, inhibit physical function decline, and alleviate depressive symptoms. This approach is hypothesized to significantly improve patients’ social life and QoL.

The following section outlines the foundation for further research on acupoint massage for MHD-induced frailty. This research will investigate clinically applicable TCM nondrug treatment strategies, providing new ideas for preventing and treating MHD-induced frailty using TCM. The intervention is low-cost and easily accessible. This combined intervention strategy is cost-effective, not venue- or equipment-specific, and has significant clinical implications.

### Expected Outcomes

It is anticipated that the frailty status of patients undergoing MHD will improve significantly, with substantial enhancement of muscle strength and lower limb function. Depressive symptoms will be alleviated, and mental health and QoL will be enhanced. Furthermore, this study will serve as a reference for effective and convenient intervention measures to further improve frailty in patients undergoing MHD.

## Methods

### Research Design

A randomized controlled trial design is used to evaluate the impact of integrating Baduanjin exercise with acupoint massage on the prevalence of frailty in patients undergoing MHD treatment.

### Participants

A total of 114 patients undergoing MHD who met the inclusion and exclusion criteria were selected from the Hemodialysis Center of the Affiliated Hospital of Chengdu University of TCM (Textbox 1).

Textbox 1. **Inclusion, exclusion, removal, withdrawal, and termination criteria for patients undergoing maintenance hemodialysis.**

**Inclusion criteria**

- Age: 45‐75 years with end-stage renal disease (uremia stage).
- Dialysis duration: receiving hemodialysis treatment ≥3 months, 3 times a week, and 4 hours per session, with stable vital signs.
- Frailty status: evaluated using the FRAIL (Fatigue, Resistance, Ambulation, Illness, and Weight Loss) scale, with scores of ≥1 indicating prefrailty or frailty.
- Cognitive and communication ability: clear consciousness, normal hearing, and ability to communicate normally.
- Physical function: no physical activity disorders, no regular exercise, able to understand and follow demonstration actions.
- Acupoint skin condition: intact skin at acupoint massage sites.
- Voluntary participation: willing to participate in the study.

**Exclusion criteria**

- Neurological disorders: presence of neurological diseases or other uncontrolled chronic diseases.
- Severe complications: severe arrhythmias, angina, severe infections, malignancies, and renal osteodystrophy.
- Noncooperation: refusal to cooperate or psychiatric abnormalities preventing collaboration.
- Concurrent clinical trials: simultaneous participation in another clinical trial.

**Removal, dropout, and termination criteria**

- Noncompliance: patients who did not follow the trial design methods or who were unable to cooperate due to illness were excluded.
- Data completeness: this was removed if the data completeness was <80% or the treatment period was less than 6 weeks.
- Voluntary withdrawal: patients who voluntarily withdrew were lost to follow-up or received a kidney transplant.
- Adverse events (AEs): the study was terminated if serious AEs or disease deterioration occurred.

### Sample Size Calculation

The sample size calculation formula for the multigroup mean is as follows:


$$n=\varphi^{2}\left(\sum_{i}s_{i}^{2}/g\right)/\left[\sum ({\bar{X}}_{l}-\bar{X}{)}^{2}/(g-1)\right]$$

where n represents the requisite sample size for each group, g represents the number of groups, and Si is the mean (SD) for each group. The values of φ are obtained from tables α, β, ν1, and ν2, with α=.05, β=.1, ν1=2, ν2=∞, and *φ*=2.52. A 2-sided test was used in this study, which stipulates that the test level α=.05, the test efficiency 1-β=.90, and the probability of a type II error is 0.10. The FRAIL scale score was used to calculate the effect size, and the average number of patients in each group, as previously described [37], was as follows: 3.29, 2.67, and 2.58, with SD values of 0.96, 0.87, and 1.02, respectively. These data were entered into the formula to calculate n=34. Considering the shedding rate of 10%, the actual required sample size for the 3 groups was estimated to be 38 per group, totaling 114 patients.

### Random Allocation Method

A randomized controlled trial design was used in this study, whereby participants were randomly allocated to the experimental and control groups. After generating the random numbers using SPSS 25.0 with the initial seed value 120983, the allocation sequence (Group A for participants 1‐38, Group B for 39‐76, and Group C for 77‐114) was concealed using sequentially numbered, opaque, sealed envelopes. These envelopes were prepared by an independent statistician not involved in participant recruitment or assessment. Each envelope contained the group assignment for the corresponding participant and was only opened by the research coordinator after a participant had completed enrollment procedures, ensuring that neither the participants nor the recruiting researchers could predict or influence group allocation. This procedure was implemented to minimize the risk of selection bias.

### Blinding

Given the nature of the interventions, blinding operators and patients was unfeasible. However, the individuals responsible for data collection and analysis were blinded to the group information to guarantee objective and impartial results.

### Intervention Measures

#### Control Group

The patient received conventional hemodialysis treatment and care without any additional interventions.

Conventional hemodialysis treatment is the standard dialysis treatment regimen that includes general symptomatic support, including the selection of appropriate dialysis machines, fluids, and methods; blood pressure and blood sugar management; correction of anemia; and prevention of secondary hyperparathyroidism.

Conventional hemodialysis care consists of 3 distinct phases. First, predialysis care was administered, where patients were introduced to the dialysis room environment, and assistance was provided in assuming a comfortable position. Dialysis machines were checked for readiness, general condition was evaluated, and predialysis weight was measured. Second, during dialysis care, we closely monitored vital signs, observed the vascular access for swelling or bleeding, ensured tight connections of external blood circuits, and responded to dialysis machine alarms. Third, postdialysis care included managing the vascular access, assessing postdialysis conditions, providing health education on diet, re-examination, and daily protection of vascular access.

#### Acupoint Massage Group

##### Overview

In addition to conventional treatment and nursing, acupoint massage intervention was conducted 3 times a week, each lasting 15 minutes. Each acupoint was massaged for about 2‐3 minutes, focusing on specific acupoints, including Zusanli (ST36), Sanyin (SP 6), Taixi (KI3), and Yongquan (KI1).

Acupoints were selected based on an extensive literature review and expert consultation using the bone-degree measurement method in conjunction with finger-inch positioning. The location of the acupoints is based on the “National Standard of the People’s Republic of China GB/T 12346‐2021 Acupoint Names and Locations” [38]. Here are the locations of the following acupoints: (1) Zusanli (ST36) located on the lateral side of the lower leg, 3 cun below Dubi (ST35), on the line connecting Dubi (ST35) and Jiaxi (ST41), (2) Sanyinjiao (SP6) located on the medial aspect of the lower leg, 3 cun above the tip of the medial malleolus at the posterior border of the tibia, (3) Taixi (KI3) located in the depression between the tip of the medial malleolus and Achilles tendon on the inner side of the ankle, (4) Neiguan (PC6, nonfistula side) located on the anterior aspect of the forearm, 2 cun above the transverse crease of the wrist on the palmar side, between the tendons of the palmaris longus and the flexor carpi radialis, and (5) Hegu (LI4, nonfistula side) located on the dorsum of the hand between the first and second metacarpal bones, approximately at the midpoint of the radial side of the second metacarpal bone.

##### Intervention Time and Frequency

Acupoint massage was administered 15 minutes before the end of dialysis [39]. Each acupoint was pressed and rubbed with the fingers for approximately 2 minutes, with 100 compressions each. The intervention was performed 3 times per week. Patient-reported sensations, such as soreness, numbness, and distension, indicated that the acupoints were accurately located. The applied pressure was maintained within the limits of patient tolerance.

##### Intervention Method

Before enrollment, the research team provided patients with a comprehensive introduction to the acupoints for pressing, including their functions and associated precautions. This ensured patient acceptance and cooperation. The acupoint pressing procedure was performed by trained members of the research team. Patients were reminded of the specific locations and functions of the acupoints during the procedure. The applied pressure was carefully adjusted to remain within the tolerance levels of the patients.

The force was applied uniformly and gently to prevent skin damage. The patient’s subjective sensations were closely monitored throughout the procedure, and the patients were observed for any signs of injury, erythema, or edema on the skin. To avoid discomfort, massage should be avoided when the patient experiences anxiety, hunger, or over-satiation.

### Sitting Baduanjin Combined With Acupoint Massage Group

In addition to conventional treatment and care, the patients received combined sitting Baduanjin exercise and acupoint massage thrice weekly. Each session comprised 30 minutes of Baduanjin exercise and 15 minutes of acupoint massage. The steps of Baduanjin are detailed in Table 1.

**Table 1.** The steps of Baduanjin.

Steps	Exercise name	Key movements and steps	Duration and frequency	Body posture tips
First step	Sit with eyes closed to calm the mind	Sit with eyes closed and back straight; fists on knees and tongue touching upper palate. Abdominal breathing and gather qi at Dantian (3‐5 min). Tap teeth 36 times and swallow saliva in 3 mouthfuls. Interlace hands to hold neck and breathe 9 times. Flick back of head 24 times (such as drumming).	3‐5 minutes (sitting quietly), 36 times (tooth tapping), 9 times (breathing), and 24 times (head flicking)	Cross-legged or legs hanging, back straight, and tongue against upper palate
Second step	Slightly swing and shake the “Tianzhu”	Interlace hands (palms up) at thigh roots. Lower head and twist neck to look left and right backward; swing shoulders with neck movement.	24 times per side	Keep upper body relaxed, coordinate neck, and shoulder movements
Third step	The “Red Dragon” stirring to produce saliva	Stir tongue in mouth (up, down, left, and right) to make saliva. Swirl saliva 36 times and swallow in 3 mouthfuls (with gurgling sound).	36 times (swirling) and 3 mouthfuls (swallowing)	Mouth closed, tongue movement gentle, and full
Fourth step	Hold breath, rub hands to warm them, and massage the back	Inhale and hold breath; rub hands until warm and massage lower back sides while exhaling. Repeat 24 times and then clench fists. Inhale and hold breath; imagine fire burning Dantian and exhale.	24 times (massage cycle) and 1 round (breath-holding and imagination)	Keep waist straight during massage and focus on dantian when imagining fire
Fifth step	Single-side “Pulley” rotation	Place 1 hand on corresponding waist (left hand on left waist and right hand on right waist). Lower head and move arm in a circle (back to front) such as a pulley.	36 times per side (left arm first and then right arm)	Arm movement smooth and keep body stable without leaning
Sixth step	Double-side “Pulley” rotation	Place both hands on waist. Lower head and move shoulders and arms in a circle simultaneously, such as a pulley. Straighten legs and rest 1 minute after movement.	36 times (circular movement) and 1 minute (rest)	Shoulders and arms move in sync and relax fully during rest
Seventh step	Cross-legged sitting with hands lifted above the head	Rub hands and exhale on palms 5 times. Interlace fingers, turn palms up, and lift hands over head; stretch waist upward. Slowly lower arms.	9 times (lifting and lowering cycle)	Sit cross-legged and stretch body fully when lifting hands and lower arms slowly
Eighth step	Lower the head and reach for the feet frequently	Palms facing each other and stretch hands forward. Bend upper body forward, grasp foot soles, and lean forward and lower head. Slowly sit up straight. After 12 repetitions: cross-legged sit, clench fists, and calm mind; swirl saliva 36 times and swallow in 6 mouthfuls.	12 times (bending and sitting up), 36 times (saliva swirling), and 6 mouthfuls (swallowing)	Bend body slowly to avoid sudden force and keep breath natural during movement

#### Intervention Schedule and Frequency

The integrated intervention protocol involved conducting sessions 3 times per week, with each session lasting 45 minutes. The duration was allocated as follows: 30 minutes for sitting Baduanjin exercises and 15 minutes for acupoint massage.

#### Intervention Methods and Preparation

A comprehensive health promotion manual focusing on sitting Baduanjin exercises and acupoint massage was developed. The manual primarily included the benefits of this integrated therapy for patients undergoing dialysis, as well as detailed implementation plans and specific techniques, exercise principles, and protocols for managing any physical discomfort that may arise.

On the day of enrollment, the sitting Baduanjin technique and associated acupoints were explained to the patients. The purpose, methodology, expected outcomes, and precautions of this integrated therapy were elaborated to ensure their understanding and cooperation. After confirming through questioning that the patients had comprehended the relevant information, the necessary materials, including forms, brochures, and a recorded video demonstrating the sitting Baduanjin practice, were distributed.

### Stage of Clinical Implementation

First, for the intervention content, a centralized intervention approach was implemented. The intervention sessions were conducted on dialysis days, 45 minutes before the start of dialysis, in the department’s health education room. Participants engaged in group practice of sitting Baduanjin exercises. Each session was supervised by 2 research team members: one demonstrated and explained the movements and breathing techniques, while the other provided real-time corrections to ensure proper form. Efforts were made to standardize movements within the participants’ physical capabilities to maximize exercise efficacy. The intervention was administered 3 times per week, with each session lasting 30 minutes and divided into 3 segments: a 3-minute warm-up focusing on joint mobility, a 25-minute main segment where sitting Baduanjin was practiced following standardized fitness qigong music instructions under the guidance of research team members, and a 2-minute cool-down period dedicated to relaxation and stretching exercises. In addition, 15 minutes before the end of dialysis, acupressure was applied to bilateral Zusanli (ST 36), Sanyinjiao (SP 6), Taixi (KI 3), and Neiguan (PC 6) on the nonfistula side, and Hegu (LI 4) on the nonfistula side. Following treatment, the participants rested for 15 minutes before leaving the dialysis room.

Second, the following precautions were taken: during the exercise regimen, if the patient experiences any of the following symptoms, which are severe fatigue, chest pain, hypoglycemic episodes, arrhythmia, shortness of breath, hypotension, or other abnormal conditions, the activity must be stopped immediately, and appropriate emergency protocols must be initiated.

As the closing form, the practitioner assumes a seated posture with their hands naturally hanging down and then adjusts their breathing.

### Adverse Event Monitoring Plan

Trained nurses recorded adverse events (AEs; hypotension, arrhythmia, etc) via real-time vital sign monitoring (every 30 minutes during dialysis) and patient-reported symptoms. AEs were graded using Common Terminology Criteria for Adverse Events (version 5.0) and reported to the principal investigator within 2 hours; severe AEs were reported to the ethics committee within 24 hours.

### Intervention Pause Criteria

Interventions were paused immediately if (1) systolic blood pressure was <90 mmHg (persistent after fluid resuscitation), (2) new-onset ventricular arrhythmia, and (3) patient-reported severe discomfort (eg, chest pain). The principal investigator would reassess within 1 hour to decide resumption or termination.

### Evaluation Indicators

#### Overview

Data collection will be conducted in strict accordance with the established schedule to ensure the integrity and timeliness of information. Research team members must be trained in standardized measurement methods to minimize measurement bias. Furthermore, baseline data (including demographic characteristics, FRAIL scale scores, grip strength, 10 times sit-to-stand (STS-10) results, Self-Rating Depression Scale (SDS) scores, and QoL (Short Form Health Survey Questionnaire [SF-36]) scores must be collected before the intervention, and follow-up data for the same indicators must be collected after 8 weeks of treatment. The specific research program, including the timing of enrollment, intervention implementation, and indicator assessment, is detailed in Table 2.

**Table 2.** Enrollment schedule, interventions, and evaluation.

Research period	Registration phase (1 week)	Divide into groups (>1 week)	Intervene (8 weeks)
Point in time			
Join a group	✓		
Eligibility screening	**✓**		
Informed consent	**✓**		
Divide into groups		**✓**	
Intervene			
Control group			✓
Routine treatment and nursing			**✓**
Routine treatment and nursing+acupressure group			**✓**
Conventional treatment and nursing+sitting Baduanjin combined acupressure			**✓**
Assessment			
FRAIL^a^ Scale score			**✓**
Grip strength			**✓**
STS-10^b^			**✓**
SDS^c^			**✓**
SF-36^d^			**✓**

a FRAIL: Fatigue, Resistance, Ambulation, Illness, and Weight Loss.

b STS-10: 10 times sit-to-stand.

c SDS: Self-Rating Depression Scale.

d SF-36: Short Form Health Survey Questionnaire.

#### Primary Evaluation Indicator (FRAIL Scale Score)

To assess the severity of frailty in patients, the scores before and after the intervention were compared as follows.

1. Decline: the FRAIL scale, a simple frailty measurement tool developed by the International Society for Nutrition and Aging in 2008 and derived from the Fried Frailty Scale, which has been widely used in different populations, is quick and easy to use and predicts the occurrence of adverse outcomes. The scale consists of 5 entries: fatigue, increased resistance or decreased endurance, decreased free mobility, disease conditions, and decreased body mass. Each entry was assigned 1 point, and the total score ranged from 0 to 5, with 0 indicating no debility, 1‐2 indicating debilitating, and ≥3 indicating debilitating. A large foreign cohort study confirmed the good predictive validity of the FRAIL scale for impairment of daily activities and the occurrence of death [40]. Compared with other debilitation measurement tools, the FRAIL scale is most closely associated with dialysis-related complications and is useful for screening for debilitation in patients undergoing MHD [41]. It was standardized by Wei et al [42], and its reliability and validity were good, with a Cronbach coefficient of 0.826 and content validity of 0.98.
2. Indicators of somatic function: due to the lack of objective indicators on the FRAIL scale, secondary evaluation indicators related to physical function were selected for measurement based on the literature review.

#### Secondary Evaluation Indicators

##### Grip Strength

Hand grip strength testing is the simplest and most widely used method for upper limb muscle strength testing. In this study, the EH101 electronic grip strength meter (Guangdong Xiangshan Company) was used to measure the grip strength. The patients were asked to hold the grip strength meter with maximum force using the noninternal fistula side of the limb in a standing position during measurement. Overall, 2 measurements were taken, with a 15-second interval between each measurement. The average value of the 2 measurements was taken.

##### STS-10

The 10 times sitting-stereotaxic position test (STS-10) is a common method for evaluating muscle strength and endurance of the lower limbs, which is easy to use, safe, and has high specificity [43]. Specific operation method where the patients were asked to cross their hands in front of their chest while sitting in the same standard-height chair so that they could withstand the speed of repeatedly standing up and sitting down 10 times. A stopwatch was used to note the time, and the 10th hip contact with the chair surface was considered the end of the exercise. A shorter time indicated better lower limb muscle strength.

##### SDS

The SDS, which was developed by Liu et al [44] in 1965, was primarily for patients’ rating of the severity of their self-depression. It is widely used in clinical studies to observe changes in patients’ depressive mood in response to interventions. The scale consists of 20 entries reflecting subjective feelings of depression, including 4 dimensions: psychotic affective symptoms, somatic disorders, psychomotor disorders, and depressive mental disorders. Each entry was scored on a 4-point scale according to the frequency of symptoms, and except for 10 entries that were scored on a 4‐1 reverse scale, the other 10 entries were scored on a 1‐4 positive scale. The total crude score was obtained by summing the entries, and the normal upper limit of the total crude score was 80, with a lower score indicating a better status. The standardized score was the integer obtained by multiplying the total crude score by 1.25. According to the Chinese normative results, the cutoff value of the SDS standardized score is 53, with no depression below 53, mild depression between 53 and 62, moderate depression between 63 and 72, and severe depression above 72.

##### SF-36

The QoL of the study participants was assessed using the SF-36, a concise health survey questionnaire developed by the Boston Health Research Institute in the United States. The Chinese version of the SF-36, developed by Prof. Jieqian Fang of Sun Yat-sen University of Medical Sciences was used in this study [45]. The scale contains 8 dimensions: vitality, physical functioning, bodily pain, general health perceptions, physical role functioning, emotional role functioning, social role functioning, and mental health, comprehensively summarizing the respondents’ QoL. The scale has 36 entries, each with a different scoring scale. Each dimension is scored as the ratio of the actual score for that dimension minus the difference between the lowest possible score for that dimension and the highest and lowest possible scores, with the ratio multiplied by a factor of 100 to give the final score for that dimension. The score for each dimension ranges from 0 to 100, and the scale total is the sum of the scores for each dimension, ranging from 0 to 800, with higher scores representing better QoL.

### Data Collection

**Figure 1.**
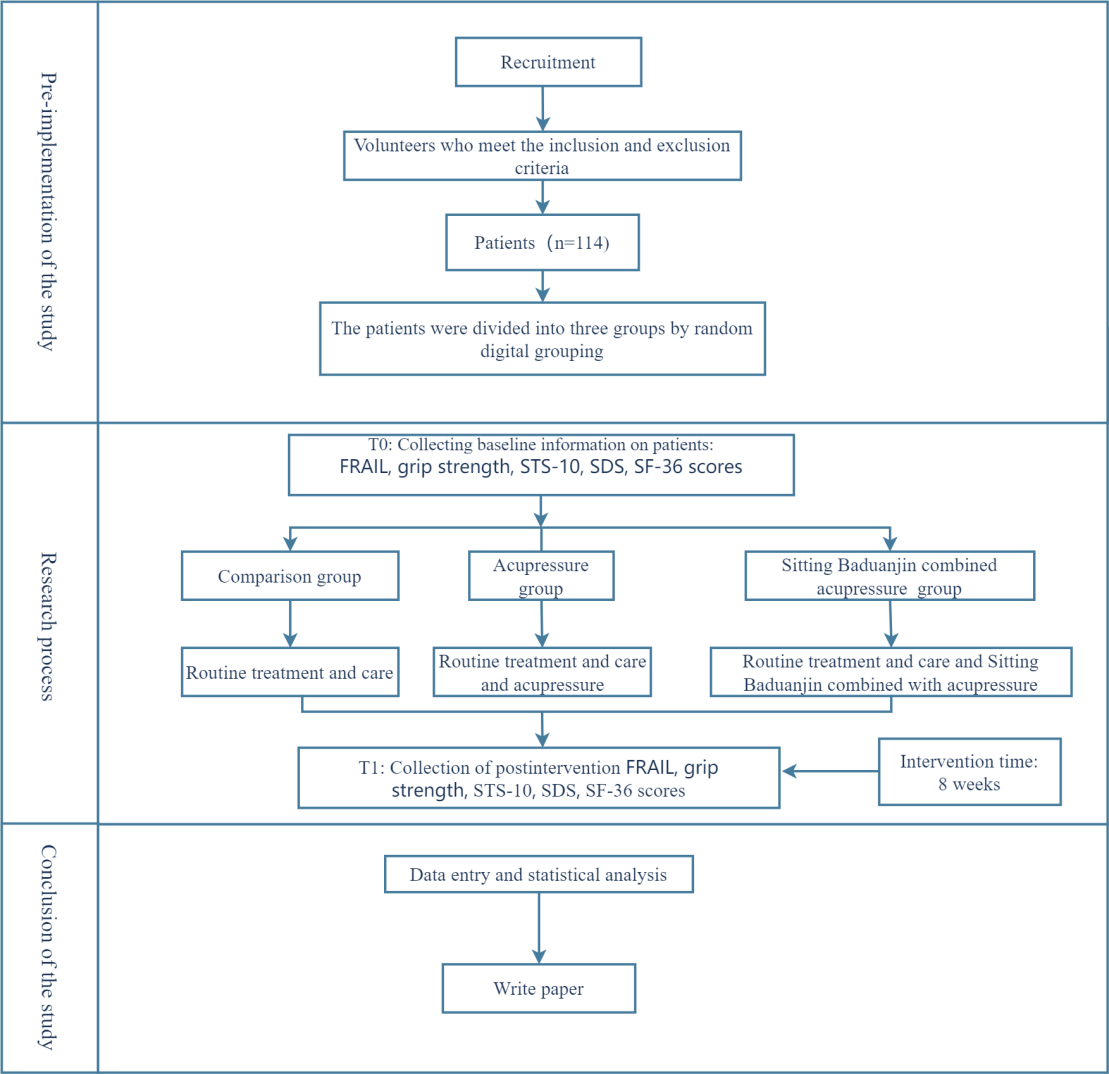
Study flowchart. FRAIL: Fatigue, Resistance, Ambulation, Illness, and Weight Loss; SDS: Self-Rating Depression Scale; SF-36: Short Form Health Survey Questionnaire; STS-10: 10 times sit-to-stand.

Research team members must be trained in standardized measurement methods. Furthermore, baseline data must be collected before and after 8 weeks of treatment. The specific research program is detailed in Figure 1.

### Quality Control

A preliminary trial plan should be developed, followed by pilot tests. It is critical to follow the established inclusion and exclusion criteria to ensure that baseline conditions across groups are balanced. Furthermore, compliance with the intervention measures and data integrity must be evaluated regularly. It is essential to provide comprehensive information regarding the advantages of the intervention, offer assistance and encouragement, and use WeChat groups for supervision to improve patient compliance.

### Statistical Methods

#### Statistical Analysis

The database was established using Microsoft Excel software, and SPSS (version 25.0; IBM Corp) was used for statistical analysis and processing of all data. A bilateral test was used for statistical analysis; *α*=.05 was used as the test level, and *P*<.05 was considered statistically significant.

#### Descriptive Statistical Analysis

The measurement sample data that conform to a normal distribution are represented as mean (SD), and the measurement sample data that conform to a skewed distribution are represented as median (IQR). The frequency and composition ratio are used to describe the data of counting samples.

#### Statistical Inference

Baseline characteristics were comparable across groups. Continuous variables were analyzed using one-way ANOVA (normally distributed data) or rank-sum test; categorical data were assessed with chi-square tests. Within-group pre-post comparisons used paired 2-tailed *t* tests (normal data) or rank-sum tests. Between-group differences postintervention were evaluated using one-way ANOVA (with least significant difference post hoc testing if significant) or rank-sum tests, as appropriate. Categorical outcomes were compared using chi-square tests.

### Ethical Considerations

The consent forms, participants’ information sheets, and all other documentation provided to the participants have received approval from the Ethics Committee of the Affiliated Hospital of Chengdu University of Traditional Chinese Medicine (2024KL-109-01). This clinical trial has been retrospectively registered with the International Traditional Medicine Clinical Trial Registry on November 12, 2024 (ITMCTR 2024000798). All participants will be thoroughly informed about the details of the clinical trial. The consent of participants will be obtained in accordance with the ethical principles and standards based on the Declaration of Helsinki. All participants will have the right to withdraw their consent voluntarily at any time, and all personal information, such as name, gender, and age, will be anonymized or deidentified to ensure that individuals cannot be identified.

Sitting Baduanjin regulates qi, blood, yin and yang, dredges meridians, and adjusts zang-fu organs. It also boasts advantages such as moderate exercise intensity, high safety, and ease of practice. It has been proven safe and effective in previous clinical trials without toxic or side effects. However, acupressure involves external force application, and some patients may experience pain or discomfort. Researchers will make every effort to prevent and treat potential harms arising from this study. If participants experience any discomfort during the research, doctors will conduct evaluations and provide medical treatment accordingly. In the event of adverse events during the clinical trial, a committee of medical experts will assess whether they are related to the trial. For trial-related injuries, researchers will cover the medical treatment costs and provide corresponding economic compensation in accordance with relevant laws and regulations.

## Results

This study was funded in December 2024, with data collection officially initiating on December 15, 2024, and projected to conclude in November 2025. As of the article submission date, 52 participants have been successfully enrolled, and all planned interventions are ongoing. Preliminary analysis of baseline demographic characteristics (eg, age, gender, dialysis duration) has been completed, confirming the balance of key variables across the 3 study groups, while full statistical analysis of intervention effects will be conducted immediately after the completion of all participant interventions and data collection. The trial is expected to be fully completed within 2025, with final study results anticipated to be published in Spring 2026.

## Discussion

### Anticipated Findings

The sitting-style Baduanjin can regulate respiration, adjust the body, and calm the mind. It can gently modulate the functions of internal organs [46]. This form of exercise is relatively less intense, easy to learn and practice, and is not constrained by location. Conversely, acupoint massage stimulates specific acupoints to regulate qi, blood, meridians, and internal organ functions. The procedure is simple and noninvasive, leading to a higher acceptance rate among patients. In addition, establishing community fitness and massage centers can reduce resource burdens and promote intervention applications.

The combination of Baduanjin and acupoint massage as a therapeutic modality within TCM is relatively less popular, and patient acceptance and preferences remain crucial factors that can influence the outcomes of this study. Beyond patient-related factors, the implementation of these TCM therapies also presents inherent complexity: both Baduanjin (the 8-section brocade) and acupoint massage require guidance and execution by individuals with specialized TCM knowledge, which necessitates substantial long-term investment in the training of skilled practitioners. For medical institutions that do not specialize in TCM, adopting and promoting these therapeutic methods thus becomes particularly challenging. Nevertheless, if health care providers and relevant stakeholders can learn and master the core theories and techniques of these TCM interventions, the benefits may extend beyond patients undergoing MHD, potentially benefiting individuals with various other chronic diseases as well.

Notably, this study has several limitations that warrant explicit acknowledgment. First, regarding the intervention duration and sample size: an 8-week intervention period may be insufficient to comprehensively elucidate the long-term therapeutic effects and patient compliance with TCM therapies. This initial time frame was determined based on the study’s research design and resource constraints, which prioritized verifying short-term safety and preliminary efficacy; however, the small sample size further impacts the robustness and generalizability of our results. Second, in terms of study design, the control group only received conventional MHD treatment and care, while the intervention group additionally received either acupoint massage alone or a combined therapy of acupoint massage and seated Qigong. This imbalance in intervention content could easily lead to disparities in attention and participation between the groups, potentially introducing biases such as placebo effects. Furthermore, the combined intervention group lacked a separate control arm (eg, a group receiving only acupoint massage or only seated Qigong), making it impossible to distinguish the independent effects of each intervention component, a gap that may weaken the strength of our evidence. Third, the nature of TCM interventions, which involve active patient participation and a visible practitioner, makes it difficult to implement blinding for both operators and patients, further increasing the risk of bias.

Adding to these limitations, the single-center nature of our sample, recruited from a specific region (eg, North China), may restrict the representativeness of our findings to diverse hemodialysis populations globally. Importantly, TCM acceptance and familiarity vary significantly across different cultural contexts: in non-Chinese settings or regions with limited exposure to TCM, patient adherence to these interventions and the subsequent therapeutic outcomes may differ substantially from those observed in our study. This cultural variability further underscores the need to address generalizability in future research.

To address these limitations and strengthen the evidence base for TCM interventions in MHD care, several future research directions are proposed. First, to improve generalizability, future studies should adopt a multicenter design involving diverse ethnicities, geographic regions, and health care systems. This will help validate whether the effects of Baduanjin and acupoint massage can be replicated across different patient populations and clinical settings. Second, conducting adaptability trials (eg, simplifying the movements of Baduanjin to enhance cultural acceptance or modifying intervention protocols to align with local health care practices) is essential for testing TCM modalities in populations with limited prior TCM exposure. Third, future research should also expand the intervention duration to assess long-term efficacy and compliance, increase the sample size to enhance statistical power, and refine the study design by including separate control arms for individual TCM components (eg, Baduanjin alone and acupoint massage alone). In addition, exploring innovative strategies to minimize bias, such as using objective outcome measures or implementing partial blinding where feasible, will further improve the rigor of future studies. Collectively, these efforts will provide a more robust validation of TCM-based interventions for patients undergoing MHD and support their potential integration into global hemodialysis care.

### Significance for Clinical Practice

The findings of this study could provide effective nonpharmacological interventions to improve frailty status, mental health, and QoL in patients undergoing MHD. This combined intervention can effectively reduce the incidence of complications and readmissions and decrease medical costs and socioeconomic pressures. In addition, it can provide new evidence for integrating TCM nursing measures into chronic disease management.

### Future Research Directions

Several strategies can be implemented to improve the reliability and comprehensiveness of studies on the effects of sit-to-stand Tai Chi combined with acupoint massage in patients undergoing MHD. First, expanding the sample size and study duration by conducting multicenter studies with larger sample sizes and extended intervention periods is essential. This would help to elucidate the long-term effects and improve generalizability accurately. Second, improving the research design by implementing stricter randomization and blinding methods is crucial to minimizing bias and ensuring the validity of the results. In addition, a comprehensive multidimensional evaluation that includes more aspects, such as social support and economic status, should be incorporated to provide a holistic view of the effects of the intervention. Third, optimizing intervention strategies by combining nutritional guidance, psychological counseling, and other relevant methods can improve the effectiveness of the intervention further. By combining these approaches, this study provides robust evidence of the efficacy of this combined TCM therapy in managing frailty in patients undergoing MHD.

### Conclusions

This study protocol presents a randomized controlled trial evaluating the efficacy of sitting Baduanjin combined with acupoint massage in improving frailty among patients undergoing MHD, addressing the gap of insufficient high-quality evidence for combined nonpharmacological interventions in this group. Its core objective is to verify whether this integrated TCM intervention alleviates frailty, enhances physical function, reduces depressive symptoms, and improves QoL, compared to routine care alone or acupoint massage alone. A key strength is the comprehensive evaluation system, integrating the FRAIL scale (primary indicator), objective physical assessments (grip strength and STS-10), and patient-reported outcomes (SDS and SF-36), alongside rigorous design (random allocation, concealed grouping, and blinded data collection and analysis), standardized protocols (8-week and thrice-weekly sessions), and detailed safety monitoring, ensuring feasibility and reliability for patients undergoing MHD. Preliminary results confirm balanced baseline characteristics across groups and good intervention safety (only mild and reversible AEs), supporting clinical applicability. If final results (anticipated Spring 2026) validate efficacy, this study will provide a scientific, low-cost nonpharmacological strategy for MHD-related frailty, enrich evidence for TCM integration into chronic kidney care, and inform clinical guidelines. Future multicenter studies with larger samples and extended follow-up are recommended to verify long-term effects and generalizability.

## Abbreviations

AE: adverse event
FRAIL: Fatigue, Resistance, Ambulation, Illness, and Weight Loss
MHD: maintenance hemodialysis
QoL: quality of life
SDS: Self-Rating Depression Scale
SF-36: Short Form Health Survey Questionnaire
STS-10: 10 times sit-to-stand test
TCM: traditional Chinese medicine

## References

[1] Shen J, Shang RR, Xue GH, Zhen RH, Liu ZH (2023). Etiological characteristics and risk factors for urinary tract infection in chronic renal failure patients undergoing MHD. Chin J Nosocomiol.

[2] Clegg A, Young J, Iliffe S, Rikkert MO, Rockwood K (2013). Frailty in elderly people. Lancet.

[3] Liu YY (2023). The relationship between frailty and quality of life in elderly maintenance hemodialysis patients: the mediating role of resilience.

[4] Fried LP, Tangen CM, Walston J (2001). Frailty in older adults: evidence for a phenotype. J Gerontol A Biol Sci Med Sci.

[5] Kurnat-Thoma EL, Murray MT, Juneau P (2022). Frailty and determinants of health among older adults in the United States 2011-2016. J Aging Health.

[6] Qin L, Liang ZZ, Ge LB (2020). Influencing factors of frailty syndrome in elderly people in the community [Article in Chinese]. Chinese General Practice.

[7] McAdams‐DeMarco MA, Law A, Salter ML (2013). Frailty as a novel predictor of mortality and hospitalization in individuals of all ages undergoing hemodialysis. J American Geriatrics Society.

[8] Takeuchi H, Uchida HA, Kakio Y (2018). The prevalence of frailty and its associated factors in Japanese hemodialysis patients. Aging Dis.

[9] Musso CG, Jauregui JR, Macías Núñez JF (2015). Frailty phenotype and chronic kidney disease: a review of the literature. Int Urol Nephrol.

[10] Bao Y, Dalrymple L, Chertow GM, Kaysen GA, Johansen KL (2012). Frailty, dialysis initiation, and mortality in end-stage renal disease. Arch Intern Med.

[11] Zhu YJ, Chen SJ, Xin X, Gao J, Liu X (2022). A study on frailty, sarcopenia, and physical functioning in maintenance hemodialysis patients. J Nurs Sci.

[12] Cai KQ, Li W, Wang L (2019). Frailty in hemodialysis patients: a survey study. J Nurs Sci.

[13] Chan GCK, Kalantar-Zadeh K, Ng JKC (2024). Frailty in patients on dialysis. Kidney Int.

[14] Cheng M, He M, Ning L (2024). Association between frailty and adverse outcomes in patients undergoing maintenance hemodialysis: a systematic review and meta-analysis. Ren Fail.

[15] Ofori-Asenso R, Chin KL, Sahle BW, Mazidi M, Zullo AR, Liew D (2020). Frailty confers high mortality risk across different populations: evidence from an overview of systematic reviews and meta-analyses. Geriatrics (Basel).

[16] Du LY, Hu X, Wang XH (2018). Prevalence of frailty in patients with maintenance hemodialysis and its effect on prognosis [Article in Chinese]. J Nurs.

[17] Wang Y, Jiang L, Ma J, Tong H, Wang L (2023). Status quo and influencing factors of symptom distress and frailty in patients who underwent maintenance hemodialysis. Chinese Evidence-Based Nursing.

[18] Soysal P, Veronese N, Thompson T (2017). Relationship between depression and frailty in older adults: a systematic review and meta-analysis. Ageing Res Rev.

[19] He MX, Yuan HH, Yang YJ, Yu S, Fu P (2022). Clinical research progress of sarcopenia and frailty in maintenance hemodialysis patients. West China Medical Journal.

[20] Garcia-Canton C, Rodenas A, Lopez-Aperador C (2019). Frailty in hemodialysis and prediction of poor short-term outcome: mortality, hospitalization and visits to hospital emergency services. Ren Fail.

[21] Li J, Xiao W, Wang L, Zhang M, Ge Y (2025). The prevalence of frailty among older adults with maintenance hemodialysis: a systematic. BMC Nephrol.

[22] Bossola M, Mariani I, Antocicco M, Pepe G, Di Stasio E (2025). Frailty, all-cause mortality, and hospitalization in patients on maintenance hemodialysis: a systematic review and meta-analysis. J Clin Med.

[23] Uslu A, Genç FZ (2025). The relationship between xerostomia, nutrition, and frailty in older patients undergoing hemodialysis. Nurs Health Sci.

[24] Chen Z, Zhu L, Ma C (2025). Frailty risk prediction models in maintenance hemodialysis patients: a systematic review and meta-analysis of model performance and methodological quality. Ren Fail.

[25] Sy J, Johansen KL (2017). The impact of frailty on outcomes in dialysis. Curr Opin Nephrol Hypertens.

[26] Mlynarska A, Mlynarski R, Golba KS (2018). Anxiety, age, education and activities of daily living as predictive factors of the occurrence of frailty syndrome in patients with heart rhythm disorders. Aging Ment Health.

[27] Tian T, Cai Y, Zhou J (2020). Effect of eight-section brocade on bone mineral density in middle age and elderly people: protocol for a systematic review and meta-analysis of randomised controlled trials. Medicine (Baltimore).

[28] Song RW, Zhang LP, Tang JH, Han M (2015). Research status of health qigong—Baduanjin adjusting psychosomatic syndrome. Inner Mongol J Tradit Chin Med.

[29] Wang YQ, Wang LB, Zheng L (2022). Effect of Ba duan Jin combined with cardiopulmonary rehabilitation training on pulmonary function in patients with stable COPD. Chin J Rehabil.

[30] Qin H, Zhang GC, Liu HH (2020). The advancement in clinical applications of the eight section brocade qigong practice. J Guangxi Univ Chinese Med.

[31] Fu JX (2023). Research on the application effect of Baduanjin exercise in patients with maintenance hemodialysis.

[32] Liu FF, Gao HM, Cui YX (2022). The synergistic effects of five-element music and eight-section brocade on fatigue and weakness in hemodialysis patients. Journal of Qi lu Nursing.

[33] Guo Q, He BY, Zhao D (2016). Influence of massage therapy on fatigue status of maintenance hemodialysis in patients. Chinese Nursing Research.

[34] Mastnardo D, Lewis JM, Hall K (2016). Intradialytic massage for leg cramps among hemodialysis patients: a pilot randomized controlled trial. Int J Ther Massage Bodywork.

[35] Ghavami H, Shamsi SA, Abdollahpoor B, Radfar M, Khalkhali HR (2019). Impact of hot stone massage therapy on sleep quality in patients on maintenance hemodialysis: a randomized controlled trial. J Res Med Sci.

[36] Li Y, Li J, Chen X, Shi Y, Shen J, Huang T (2024). What specific exercise training is most effective exercise training method for patients on maintenance hemodialysis with sarcopenia: a network meta-analysis. Front Nutr.

[37] Tsay SL (2004). Acupressure and fatigue in patients with end-stage renal disease-a randomized controlled trial. Int J Nurs Stud.

[38] National Standard of the People’s Republic of China (2020). Name and Location of Meridian Points (GB/T12346-2021).

[39] Junjun G (2019). The study of acupuncture point massage intervention on fatigue of hemodialysis patients.

[40] Lopez D, Flicker L, Dobson A (2012). Validation of the frail scale in a cohort of older Australian women. J Am Geriatr Soc.

[41] Chao CT, Hsu YH, Chang PY (2015). Simple self-report FRAIL scale might be more closely associated with dialysis complications than other frailty screening instruments in rural chronic dialysis patients. Nephrology (Carlton).

[42] Wei Y, Cao Y, Yang X (2018). A study on the Chineseization and reliability of a screening tool for debilitating risk in elderly inpatients. Chinese Journal of Practical Nursing.

[43] Segura-Ortí E, Martínez-Olmos FJ (2011). Test-retest reliability and minimal detectable change scores for sit-to-stand-to-sit tests, the six-minute walk test, the one-leg heel-rise test, and handgrip strength in people undergoing hemodialysis. Phys Ther.

[44] Liu X, Dai Z, Tang M (1994). Factor analysis of the Self-Depression Scale (SDS) results for medical students [Article in Chinese]. Chin J Clin Psychol.

[45] Ji-qian F, Chonghua WAN, Mingli SHI (2000). Overview of quality of life studies and measurement scales. Modern Rehabilitation.

[46] Xingxing X, Xiaowen Z, Ying X (2023). Effect of sitting baduanjin combined with acupoint injection on quality of life in patients with acute exacerbation of COPD. Wisdom of Health.

